# Unusual Presentation of Hemophagocytic Lymphohistiocytosis in a Kidney Transplant Patient

**DOI:** 10.1155/2019/3682378

**Published:** 2019-03-10

**Authors:** Danwen Yang, Natanong Thamcharoen, Chelsea Marcus, Andreas Varkaris, William Aird, Eliyahu V. Khankin, Francesca Cardarelli

**Affiliations:** ^1^Division of Nephrology, Beth Israel Deaconess Medical Center, Harvard Medical School, Boston, MA, USA; ^2^Division of Pathology, Beth Israel Deaconess Medical Center, Harvard Medical School, Boston, MA, USA; ^3^Division of Hematology, Beth Israel Deaconess Medical Center, Harvard Medical School, Boston, MA, USA

## Abstract

We are presenting a case of a middle-aged woman with history of remote kidney transplantation who had multiple admissions for septic shock-like picture, recurrent fever, and hypotension. Her shock manifestation would resolve after stress dose steroid administration and less than 24 hours of vasopressor administration. Initially, extensive workup was performed without revealing etiology. Eventually, a bone marrow biopsy was carried out leading to the diagnosis of hemophagocytic lymphohistiocytosis, most likely related to recent cytomegalovirus infection.

## 1. Introduction

Hemophagocytic lymphohistiocytosis (HLH) is a life-threatening syndrome resulting from excessive immune activation. It usually presents as a febrile disease progressing to multiple organ failure. If HLH is not promptly recognized and treated, it is associated with a high mortality rate, often within months of initial symptoms. Primary HLH is a genetic disease, while acquired (or secondary) HLH occurs mostly in the setting of infections, malignancies, or autoimmune disease. Here, we present the case of a middle-aged female who developed HLH during the recovery phase of a cytomegalovirus (CMV) infection, 23 years after kidney transplantation. Our patient had an atypical and subacute manifestation, likely due to blunted immune response resulting from chronic immunosuppression. This presented a substantial challenge in prompt diagnosis of the condition.

## 2. Case

A 63-year-old female with history of end stage renal disease secondary to IgA nephropathy, who underwent a living related kidney transplantation in 1995, presented to our hospital with generalized malaise, dyspnea on exertion, and cough which started 6 months prior. Her other past medical history included type 2 diabetes mellitus and chronic kidney allograft dysfunction due to recurrent IgA nephropathy. Immunosuppressive regimen included cyclosporine 100 mg every 12 hours, azathioprine 50 mg daily, and prednisone 5 mg daily. On arrival to the hospital, the patient was hypotensive with a blood pressure of 75/48 mmHg and febrile with a temperature of 100.6 F. Norepinephrine drip and broad-spectrum antibiotics were initiated, although a source of infection was not obvious at that time. Laboratory testing was notable for anemia, thrombocytopenia, and elevated lactate dehydrogenase (LDH) and C-reactive protein (CRP). CMV viral load was positive with a titer of 3.6 log10 IU/ml and valganciclovir therapy was initiated as a result. Morning cortisol level was substantially suppressed at 0.6mcg/dL, and, due to concern for adrenal insufficiency, stress dose hydrocortisone was administered, followed by conversion to prednisone taper. Patient's hypotension and fever resolved within 24 hours from the initial presentation, and she was subsequently discharged in stable condition with a diagnosis of CMV infection, on appropriate dose of valganciclovir with plan for follow-up as an outpatient. Unfortunately, the patient was rehospitalized 4 times in the subsequent 2 months with fever, shock, and fatigue. During each admission she received stress dose hydrocortisone and vasopressor with or without empiric antibiotics. Every time, her symptoms resolved rapidly (within 24 hours of initiation of therapy) without a clear diagnosis. Initially CMV was considered to be the cause of recurrent fever and hematologic abnormalities, but she had recurrent severe symptoms despite resolution of CMV viremia in the setting of valganciclovir treatment (summary in Table 1).

**Figure 1:**
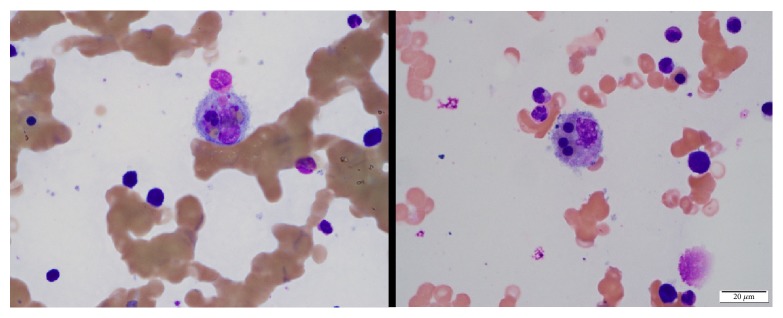
Bone marrow aspirate demonstrating hemophagocytic histiocytes with engulfed nucleated erythroid precursors (right) and platelets and neutrophils (left).

On the 5th admission, she presented again with hypotension and fever. The remainder of a review of symptoms was negative. Physical examination was unremarkable. Laboratory testing revealed a white blood cell count of 7,200/mm^3^, persistent anemia with a hemoglobin of 8.2 g/dl, and thrombocytopenia with a platelet count of 83,000/mm^3^. LDH and CRP were yet again elevated (410 IU/L and 153.0 mg/L, respectively). Alanine aminotransferase and aspartate aminotransferase were mildly elevated at 50 IU/L and 41 IU/L, respectively. The patient's serum triglyceride level was 547 mg/dl (normal range 0–149 mg/dl) and ferritin level was remarkably elevated at 3311 ng/ml (normal range 13–150 ng/ml). As outlined above, CMV viral load was undetectable. Vasopressor and stress dose hydrocortisone were administered. As patient's hypotension resolved, vasopressor was discontinued within 24 hours from the time of admission. A computed tomography scan of the chest, abdomen, and pelvis showed patchy ground glass opacities at the lung apices bilaterally. Bronchoscopy with broncho-alveolar lavage was performed, though it did not reveal any evidence of infection, including* Pneumocystis jirovecii*. Further testing was performed to exclude rheumatologic disease, macrophage activation syndrome, malignancy, and autoinflammatory syndromes. Complement levels were within normal limits; antinuclear antibodies and rheumatoid factor resulted as negative. Serum protein electrophoresis test showed normal levels of all immunoglobulins and no monoclonal component. EBV viral load was negative. Patient inevitably underwent a bone marrow biopsy, which revealed histiocytes with engulfed red cells, platelets, and neutrophils (Figure 1), suggesting HLH. High dose intravenous methylprednisolone was started at 1 gram intravenously daily and continued for 5 days, after which she was continued on oral prednisone at 1 mg/kg/day. Soluble Interleukin 2 receptor (sIL-2R) was elevated (15490 pg/ml, normal range <1033). Based on this, patient was diagnosed with HLH and discharged on high dose oral prednisone.

She remained afebrile, but suffered from severe persistent weakness. Subsequent outpatient laboratory testing revealed reduction of the platelet count and increased ferritin, indicating ongoing macrophage activation. Patient underwent thorough and extensive genetic testing, which was not consistent with familial HLH. Torso imaging was repeated to rule out an underlying malignancy, with positron emission tomography, and a magnetic resonance imaging did not reveal any suspicious neoplastic lesion. Given clinical and laboratory signs concerning for HLH exacerbation, chemotherapy with etoposide was started. She received four doses of etoposide in combination with daily dexamethasone. Unfortunately, patient developed acute hypoxic respiratory failure due to aspiration pneumonia in the setting of progressive encephalopathy, combined with* Enterococcal* and* Neisseria* bacteremia, and ultimately passed away within 3 months of her initial presentation.

## 3. Discussion

HLH involves defective natural killer T cell function, resulting in aberrant activation of cytokine cascade and hemophagocytosis in the bone marrow. This can present as refractory prolonged fever and hepatosplenomegaly and eventually lead to multiorgan failure, and death [1]. Common laboratory findings include cytopenias, elevated ferritin, low or absent natural killer (NK) cell activity, elevated sIL-2R, hypertriglyceridemia, and low fibrinogen. Specific diagnostic criteria have been developed by the Histiocyte Society (HS) trials, HLH-94 and HLH-2004, and currently 5 of 8 criteria are required to make a diagnosis of HLH [2]. Based on fever, cytopenia, elevated ferritin, hypertriglyceridemia, elevated sIL-2R, and hemophagocytosis observed on the bone marrow biopsy, our patient met the diagnostic criteria for HLH. Limitations of these criteria are related to the fact that they have not been validated in adults, or in the reactive form of the syndrome. HScore is the first validated score system devoted to the diagnosis of reactive HLH. In the validation set, the median HScore was 222 (interquartile 202–284) for positive cases and 129 (interquartile 77–152) for negative cases. In our case, the HScore was 228, which is associated with >97% probability of HLH [3].

According to the underlying etiology, HLH can be classified into either primary or secondary form. Primary HLH is associated with genetic immunodeficiency syndromes [4]. In acquired HLH, abnormal lymphocyte T cells are activated by triggers such as infections, malignancies, and autoimmune diseases, which further stimulate macrophages. Activated macrophages engulf or phagocytose erythrocytes, lymphocytes, and other hematopoietic precursors, causing pancytopenia and organomegaly. The main triggers of secondary HLH are viruses (35%), bacteria (9%), fungi (2%), and parasites (2%) [5]. Epstein-Barr Virus (EBV) is a common infectious trigger for secondary HLH [6]. One study in patients with EBV-associated HLH found an association between viral load levels and disease activity [7].

Since 1979, 21 cases of CMV-associated HLH in kidney transplant recipients were reported in the literature [8]. In the majority of cases, HLH occurred within 1 and 6 months after transplantation, and it represented a reactivation of a latent disease. Mortality was reported in 9 out of 21 cases (42.8%) [8]. For cases with an available detailed medical history, progression to lethal outcome was quite rapid, ranging from 9 to 21 days. CMV-associated HLH in the late posttransplant phase, i.e., more than 2 years after kidney graft implantation, to our knowledge, has never been described to date. Here we report the first case of CMV-related HLH, which developed 23 years after kidney transplantation and presented with a subacute and atypical picture, making it a very challenging diagnosis.

This emphasizes the conclusion that HLH should be suspected in any kidney transplant patient experiencing sepsis-like symptoms without a clear infectious cause, and regardless of the time after transplantation. Furthermore, it should be noted that HLH can have variable presentations, as in our patient, who had a subacute (and at times indolent) course. We believe that it should be a likely assumption that blunted clinical presentation has been a result of exposure to chronic immunosuppressive regimen. Though previous studies showed that high viral load positively correlated with the development and prognosis of HLH, our patient had progressive and persistent HLH despite clearance of CMV viremia in plasma, though it is plausible that our patient had CMV infection in other compartments, which could have caused persistent HLH, while viral load in plasma remained negative. However, she had no gastrointestinal or upper respiratory tract symptoms to suggest this was the case.

The treatment of HLH is based on the severity of the disease. Importantly, the treatment should be started as soon as HLH is suspected, given the high morbidity and mortality. Detection of an underlying genetic defect is important, since it changes the management substantially with main focus on early search for a stem cell donor, which is best recommended approach. Current HLH therapies include suppression of the inflammation with corticosteroids, cyclosporin A, IVIG, and/or anticytokine agents. Activated immune cells can also be the target of medications like etoposide, rituximab, and/or antithymocyte globulin. Identified causes of HLH, like infections and malignancies, must be treated. Finally, if the HLH is a primary disease, stem cell transplantation should be considered [9].

## 4. Conclusion

The clinical features of HLH can be nonspecific and overlap with a variety of other illnesses such as sepsis, liver failure, or multiple organ dysfunction. This range of differential diagnoses makes this disease difficult to identify. HLH secondary to CMV is a rare but life-threatening condition. Although it has been described in early post-kidney transplant recipients with CMV infection, to our knowledge it was not yet reported in patients more than two years after kidney transplantation. We report the first case of HLH, which occurred many years after kidney transplantation with an atypical presentation of recurrent and transient shock episodes during the recovery phase of CMV infection. This atypical presentation was likely due to a blunted immune response in the setting of chronic immunosuppressive graft maintenance therapy. HLH requires prompt diagnosis and appropriate therapy as the mortality risk is extremely high. A diagnosis of HLH should be considered in a transplant patient presenting with recurrent fevers and shock episodes not related to clear causes, especially in the presence of unexplained anemia and thrombocytopenia, in the setting of CMV infection.

## Figures and Tables

**Table 1:** Summary of hospitalizations.

Admission	Clinical features	Discharge Diagnosis
1st (Day 1-5)	Fever and hypotension, which required norepinephrine for few hours, and responded to stress dose hydrocortisone. She became afebrile and hemodynamically stable within 24 hours.	CMV infection (CMV viral load 3.6 log⁡10 IU/ml), Epstein-Barr Virus (EBV) detectable at 282 copies/ml. She was discharged on valganciclovir, and azathioprine was discontinued.

2nd (Day 17-20)	Fever and mild hypotension, which resolved with IV fluid.	CMV infection with improving CMV viremia. Valganciclovir was continued.

3rd (Day 40-42)	Hyperglycemia.	New onset diabetes mellitus. The patient was started on insulin. CMV viral load became undetectable, but valganciclovir was continued.

4th (Day 44-49)	High grade fever and hypotension, which required norepinephrine for few hours, and responded to stress dose hydrocortisone. The patient became afebrile and hemodynamically stable within 24 hours.	Adrenal insufficiency. CMV viral load was undetectable, valganciclovir was continued.
